# In Vitro Evaluation of Potentially Edible Brazilian Trees and Shrubs in Ruminant Nutrition

**DOI:** 10.3390/ani13233703

**Published:** 2023-11-29

**Authors:** Jozivaldo Prudêncio Gomes de Morais, Mariana Campana, Pablo Gregorini, Thainá Moreira Garcia, Júlia Franco de Aguiar Minussi, Stela Naetzold Pereira, Fabiellen Cristina Pereira, Tiago Antonio Del Valle

**Affiliations:** 1 Centro de Ciências Agrárias, Universidade Federal de São Carlos, Araras 13600-970, Brazil; jozivaldo@ufscar.br (J.P.G.d.M.); mariana.campana@ufscar.br (M.C.); thainagarcia@estudante.ufscar.br (T.M.G.); juliaminussi@uol.com.br (J.F.d.A.M.); 2 Department of Agricultural Sciences, Lincoln University, Lincoln 7647, New Zealand; pablo.gregorini@lincoln.ac.nz (P.G.); fabiellen.pereira@lincolnuni.ac.nz (F.C.P.); 3 Department of Animal Science, Rural Sciences Center, Federal University of Santa Maria, Santa Maria 97105-900, Brazil; snaetzold@gmail.com

**Keywords:** Aroeira, Candeia, digestibility, Gliricídia, Jatobá, mulberry, nutritional value, methane, Santa Bárbara tree, Tithonia

## Abstract

**Simple Summary:**

Edible trees and shrubs have been evaluated to improve animal nutrition and mitigate the emission of greenhouse gasses from ruminants. In the present study, researchers visited some farmers and identified eight potentially edible Brazilian trees. Nutritional value and gas (and methane) production from these edible trees were evaluated. The leaves of Aroeira, Candeia, and Jatobá had limited nutritional value. Samples of Santa Bárbara trees, Mulberry, and Tithonia showed an improved nutritional value. Furthermore, Tithonia decreased methane emissions and is a potentially edible tree for multifunctional redesigned ruminant production systems.

**Abstract:**

The present study aims to evaluate the nutritional value of different tree and shrub leaves in Brazilian ruminant production systems. Eight potentially edible trees and shrubs were identified from interviews with 30 ruminant producers: Aroeira (ARO; *Lithraea molleoides*), Black Mulberry (BMU; *Morus nigra*), Candeia (CAN; *Eremanthus erythropappus*), Jatobá (JAT; *Hymenaea courbaril*), Gliricídia (GLI; *Gliricidia sepium*), Santa Bárbara tree (SBT; *Mélia azedarach*), Tithonia (TIT; *Tithonia diversifolia*), and White Mulberry (WMU; *Morus alba*). Four leaf samples of each edible tree were sampled, and chemical analyses and in vitro assays were performed. Edible trees (except CAN and JAT) had lower neutral detergent fiber content than Mombasa grass. In addition, SBT, BMU, WMU, and TIT had lower fiber content than the other evaluated edible trees. Consequently, SBT, TIT, BMU, and WMU had improved dry matter degradation. Among the edible trees and shrubs, SMW and WMU increased the potential for gas production (a parameter). On the other hand, CAN decreased the estimated gas production 48 h after incubation. Furthermore, TIT decreased methane production up to 24 h after in vitro fermentation. Thus, except ARO, CAN, and JAT, the edible trees evaluated in the present study are potential feeds in moderate- to high-producing animals. Additionally, TIT fermentation reduces in vitro methane production.

## 1. Introduction

Climatic change is one of the main challenges humanity faces nowadays. It negatively affects animal production and increases food security risks [1]. Ruminant production systems have been redesigned using multifunctional concepts [2] to attenuate climatic changes. Trees have been inserted in these redesigned systems due to multiple ecosystem services, such as improving soil fertility, limiting soil erosion, and regulating the climate with CO_2_ sequestration [2].

Tropical forages generally have low protein and high fiber content, which decreases animal production efficiency [3]. In this situation, edible trees and shrubs can produce a high volume of biomass with good nutritional value in local soil and climatic conditions [2]. Some of these materials (for example, mulberry [4], Gliceridia [5], and Tithonia [3]) have high protein and soluble carbohydrate content and are potential feed in ruminant nutrition, especially in sustainable farms. In addition, including tropical trees and leaves in the diet of ruminants can reduce methane emissions by 10–25% [6].

Although several questions, such as leaf regrowth after grazing and animal intake, need to be considered [4,6], it is essential to evaluate the nutritional potential of different edible trees and shrubs [7]. Therefore, we hypothesize that edible trees and shrubs have reduced fiber content, higher protein content and in vitro degradation, and lower methane production than traditional forage sources (corn silage and *Megathyrsus maximus* cv. Mombasa). Thus, these plants could be recommended in the future for the redesign of productive landscapes. This study aims to evaluate the chemical composition, in vitro degradation, and in vitro gas production of different edible trees and shrubs.

## 2. Materials and Methods

The present trial was performed at the Centro de Ciências Agrárias of Universidade Federal de São Carlos (UFSCar, Araras, São Paulo State, Brazil) from July 2022 to May 2023. All procedures using animals were previously approved by the UFSCar Ethics Committee (approval number 9582200722).

### 2.1. Edible Tree and Shrub Identification and Sampling

Thirty small farms were chosen from an institutional extension program. The following criteria were considered: (I) worked with cattle production and (II) was located near the university (less than 100 km). Farms were visited and producers’ interviews were conducted to identify local edible trees or shrubs. The average age of the interviewed producers was 73 years old. The following inclusion criteria of trees and shrubs were (I) local availability and (II) animal eating observation. Eight potential edible trees and shrubs were identified: Aroeira (ARO; *Lithraea molleoides*), Black Mulberry (BMU; *Morus nigra*), Candeia (CAN; *Eremanthus erythropappus*), Jatobá (JAT; *Hymenaea courbaril*), Gliricídia (GLI; *Gliricidia sepium*), Santa Bábara tree (SBT; *Melia azedarach*), Tithonia (TIT; *Tithonia diversifolia*), and White Mulberry (WMU; *Morus alba*). In addition, whole-plant corn silage and Mombasa grass (*Megathyrsus maximus*) samples were obtained in the same localities to serve as the control. Corn silage samples were collected from the exposed face of bunker silos. Mombasa grass samples were obtained by grazing simulation in pastures managed at 50 to 100 cm height.

### 2.2. Chemical Analysis

Four farms were defined as independent experimental units to obtain samples to evaluate the chemical composition and in vitro degradation and gas production. Almost 500 g of leaves (silage and pasture) were sampled from each experimental unit from 27 June to 7 December 2023. Samples (*n* = 40) were dried in an air-forced oven at 60 °C for 72 h. Then, samples were processed in a knife mill equipped with a 2 mm sieve (in vitro assay) and a 1 mm sieve (chemical composition analysis). Samples were analyzed for dry matter (DM; method 930.15), organic matter (1000—ash; 942.05), and crude protein (CP; method 954.01) according to AOAC (2000) [8]. Neutral-detergent fiber (NDF) and acid-detergent fiber (ADF) were analyzed using the method in [9], using alpha amylase and without sodium sulfite. The total of phenolic compounds was evaluated in a composite sample from each edible tree using the method in [10]. Briefly, 500 mg of the sample was homogenized with 5 mL of distilled water and a methanol solution (1:1). The solution was centrifuged (500× *g* for 15 min at 10 °C) and readings were performed in a spectrophotometer at 720 nm.

### 2.3. In Vitro Assay

In vitro degradation was evaluated using the method in [11] and a NL162^®^ fermenter (NewLab, Piracicaba, Brazil). The samples (2 mm processed) were placed in 5 × 5 cm bags made of non-woven fabric (100 g/m^2^) [12]. The bags were incubated at 39 °C for 48 h in vials containing 2.0 L of an inoculum (1.6 L of [13] buffer and 0.4 L of fresh ruminal fluid). Ruminal fluid was obtained from two Holstein heifers (600 kg of body weight), who were maintained in a *Megathyrsus maximus* pasture without concentrate supply and had free access to a mineral mix. After incubation, the bags were washed in running water and analyzed for NDF, as previously described. In vitro degradation was calculated considering DM and NDF disappearance between incubation and final weight.

In vitro gas production was evaluated using 100 mL glass bottles (three for each sample) and the following procedure in [14]. The ruminal fluid was obtained from the same two Holstein heifers and maintained at 39 °C until the assay began. The samples were weighed (1 g DM) in filter bags (non-woven fabric, 100 g/m^2^) and incubated with 50 mL of a [13] buffer (40 mL) and ruminal fluid (10 mL). The bottles were sealed with a rubber stopper and incubated at 39 °C for 72 h. Gas production was recorded 6, 12, 18, 24, 30, 36, 48, and 72 h after incubation using syringes. Accumulated gas production through the in vitro assay was evaluated as repeated measures. At the 24 h evaluation, gas was sampled and analyzed for methane content using gas chromatography [15]. At the end of the incubation, inoculum pH was evaluated using a bench pHmeter (MB-10, Marte, Santa Rita Sapucaí, Brazil).

### 2.4. Calculations and Statistical Analysis

**Figure 1:**
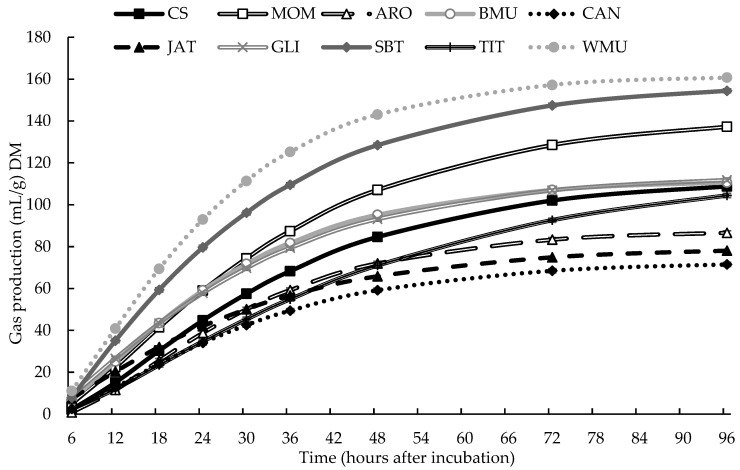
Gas production during the in vitro assay of leaves from edible trees and shrubs in Brazil.

The accumulated gas production (*GP*, mL/g dry matter) was studied using a non-linear model [16], as follows:

$$GP=A\times \left(1-e^{\left[-b\times \left(t-T\right)-c\times (\sqrt{t}-\sqrt{T})\right]}\right)$$

where *A* is potential gas production (mL/g dry matter); *b* and *c* are parameters of fractional gas production; *t* is actual incubation time (h); and *T* is the lag time (h). Considering the parameters of each treatment, gas production at 48 h of incubation and during the entire assay was estimated (Figure 1).

Data were analyzed using PROC MIXED of SAS (9.4./Viya 3.5 version, SAS Inc., Cary, NC, USA, 2022) and the following model:

*Y_ij_* = *µ* + *SP_i_* + *e_ij_*

with $e_{ij}\approx N\left(0,\sigma_{i}^{2}\right)$, where *Y_ij_* is the observed value of the dependent variable; *µ* is the overall mean; *SP_i_* is the fixed effect of sample specie; *e_ij_* is the random error; *N* stands for Gaussian distribution; and $\sigma_{i}^{2}$ is the residual variance of each specie. Species effects were studied using Fisher’s means test (LSD) at 5% of probability.

## 3. Results

### 3.1. Chemical Composition

Among the edible trees and shrubs evaluated in the present study, CAN showed the highest (*p* ≤ 0.05) DM content (Table 1). On the other hand, TIT had the lowest (*p* ≤ 0.05) DM content. The leaves of ARO had the highest (*p* ≤ 0.05) OM content, whereas BMU, TIT, and WMU had the lowest (*p* ≤ 0.05) OM content. All edible trees (except CAN) had lower (*p* ≤ 0.05) NDF content than MOM.

Santa Barbara Tree, BMU, WMU, and TIT had lower (*p* ≤ 0.05) fiber content than the other evaluated edible trees. The leaves of ARO, JAT, and GLI had an increased (*p* ≤ 0.05) ADF to NDF ratio compared to other edible trees and shrubs leaves. The highest CP content was observed (*p* ≤ 0.05) in SBT, TIT, and WMU.

### 3.2. In Vitro Degradation and Gas Production

The leaves of mulberry (BMU and WMU) and TIT had improved (*p* ≤ 0.05) DM in vitro degradation than the other edible trees (Table 2). In the opposite sense, ARO and CAN had the worst (*p* ≤ 0.05) NDF degradation between the evaluated edible trees. During the in vitro assay, JAT and TIT had increased (*p* ≤ 0.05) pH compared to traditional forage sources (CS and MOM). In addition, among the edible trees and shrubs, SMW and WMU increased (*p* ≤ 0.05) the potential of gas production (a parameter). On the other hand, CAN significantly decreased (*p* ≤ 0.05) the estimated gas production 48 h after incubation. However, treatments evaluated in the present study showed a similar (*p* ≥ 0.387) fraction of gas production (b and c parameter) and lag time (T). Furthermore, TIT decreased (*p* ≤ 0.05) the methane production up to 24 h after in vitro fermentation. On the other hand, SBT and WMU had higher (*p* ≤ 0.05) methane production compared to ARO, BMU, CS, and MOM.

Estimated gas production through the in vitro assay is showed in Figure 1, considering the parameter observed in Table 2.

## 4. Discussion

We hypothesized that edible trees and shrubs have a better chemical composition and higher in vitro degradation and lower gas production than traditional forage sources (corn silage (CS) and mombasa grass (MOM)). In general, the edible trees had lower fiber content (except Candeia (CAN) and Jatobá (JAT)) and higher DM degradation (except CAN and Aroeira (ARO)). In addition, CAN, JAT, and Tithonia (TIT) decreased gas production, whereas TIT decreased methane production.

In general, the leaves of the edible trees had lower fiber content than CS and MOM. It is essential to consider that samples are structurally different. Tithonia had leaves, leaflets, and rachis, whereas other edible tree samples were composed exclusively of leaves. On the other hand, traditional forage samples were composed of whole plants, with a high proposition of stems. According to [17], higher cellular wall content and lower digestibility of stems in relation to leaves are well documented. It is essential to highlight that trees and shrubs introduced in more diverse grazing environments allow animals to select leaves instead of traditional forage sources. Therefore, these comparisons make practical sense.

Candeia and JAT had similar fiber content when compared to traditional forage sources. Candeia had the highest DM content. This is a tree from Brazilian Savana. These trees are adapted to low water availability and reduce leaves’ water content and increase fiber concentration to avoid water losses. To the best of our knowledge, there is no study evaluating CAN as an energy and fiber source in ruminant diets. However, [18] reported that CAN essential oils have been associated with acaricidal activity.

In the present study, SBT, BMU, WMU, and TIT had lower fiber content than the other evaluated edible trees. In addition, the highest CP content was obtained from SBT, TIT, and WBU. In evaluating chemical composition and in vitro degradation of different edible trees, [19] observed that SBT (*M. dubia*) had 308 g/kg NDF and 165 g/kg CP content and 0.881 of in vitro dry matter degradation, which reinforces the high nutritional value of SBT. The high nutritional value of mulberry (BMU and WMU) leaves has been well documented [20,21]. The authors of [21] observed 206–213 g/kg NDF and 193 to 200 g/kg CP in different cultivars of mulberry. Furthermore, TIT had 395 g/kg NDF, which is in accordance with the 326 to 418 g/kg range observed by [22]. A higher CP content is related to a decreased fiber content and provides a substrate for microbial growth, improving ruminal degradation [23].

Between the evaluated edible trees, ARO, JAT, and GLI showed the highest ADF to NDF ratio. Aroeira is an Anacardiaceae, whereas JAT and GLI are from the Fabacea family. The leguminous trees had a higher ADF to NDF ratio compared to grasses. As reported in [23], a higher ADF to NDF ratio is associated with fiber fragility and digestibility, positively affecting animals’ feed intake. However, these edible trees did not show improved in vitro degradation in the present study.

Mulberry (BMU and WMU) and TIT increased in vitro degradation compared to other edible trees. As previously discussed, this improved DM degradation is associated with lower fiber content observed in these edible tree samples. In addition, improved DM and NDF degradation of edible trees may be associated with the presence of polyphenols in the plants. As reported by [4], the flavonoids present in the leaves can be used as an alternative source of carbon for the metabolism of the ruminal microbiome, as they are readily degraded in the rumen to produce non-aromatic fermentation products.

The worst NDF degradation estimate was observed in ARO and CAN. According to [24], Aroeira (*Schinus molle*) has more than 50 g/kg DM of tannins. In addition, CAN has been used as a tannin source in the industry [25]. In the present study, ARO, CAN, and JAT had 75.5 ± 0.62, 37.0 ± 1.11 and 27.5 ± 0.50 g/kg of total phenolic compounds. Other edible trees had less than 19 g/kg of phenolic compounds. Tannins are known to reduce rumen degradation of dry matter and inhibit gas production [26]. According to [26], tannins interact with protein and inhibit rumen fibrolytic microbe, reducing cell wall degradation. Therefore, CAN was one edible tree with lower gas production during the current assay. In addition, JAT and TIT showed decreased gas production. Reduced gas production in JAT could be associated with decreased in vitro degradation, which limited the substrate for gas production.

Tithonia significantly decreased methane production during the current in vitro assay study. Tithonia has methane-reducing compounds, such as condensed tannins, essential oils, and saponins [27]. Evaluating TIT addition in dual-purpose cows in pasture conditions, [28] observed a 6.8% reduction in methane emission (from 223.2 to 208.05 g/animal/day) using TIT at 15% of pasture.

## 5. Conclusions

Aroeira, Candeia, and Jatobá have limited nutritional value. However, black mulberry, Gliricídia, Santa Bárbara tree, Tithonia, and white mulberry are potential feeds in moderate- to high-producing ruminants. These edible trees have lower fiber and higher in vitro degradation than traditional forage sources. Additionally, TIT fermentation reduces in vitro methane production. Studies evaluating the effect of TIT on animal feed intake, performance, and greenhouse gas emissions are relevant.

## Figures and Tables

**Table 1:** Chemical composition of leaves from edible trees and shrubs in Brazil.

Item	Species ^1^										*p* ^2^
	CS	MOM	ARO	BMU	CAN	JAT	GLI	SBT	TIT	WMU	
DM ^3^	278 ^b^ ± 14.6	243 ^b^ ± 10.4	363 ^b^ ± 20.5	301 ^b^ ± 24.6	868 ^a^ ± 12.1	258 ^b^ ± 17.0	247 ^b^ ± 17.0	308 ^b^ ± 22.0	154 ^c^ ± 11.4	235 ^bc^ ± 20.8	<0.001
OM ^4^	937 ^ab^ ± 17.4	911 ^b^ ± 3.6	945 ^a^ ± 1.0	886 ^c^ ± 10.2	937 ^b^ ± 0.9	922 ^b^ ± 5.6	911 ^b^ ± 8.7	905 ^b^ ± 6.5	874 ^c^ ± 3.9	875 ^c^ ± 7.1	<0.001
NDF ^5^	619 ^b^ ± 54.6	762 ^a^ ± 13.5	431 ^c^ ± 8.4	322 ^d^ ± 23.9	672 ^ab^ ± 17.7	595 ^b^ ± 25.5	380 ^cd^ ± 22.9	277 ^e^ ± 20.2	395 ^cd^ ± 35.5	331 ^d^ ± 16.1	<0.001
ADF ^6^	284 ^abc^ ± 49.7	333 ^b^ ± 7.2	298 ^b^ ± 15.9	128 ^d^ ± 11.3	320 ^b^ ± 18.2	388 ^a^ ± 10.8	231 ^c^ ± 21.4	128 ^d^ ± 6.4	131 ^d^ ± 9.2	132 ^d^ ± 10.7	<0.001
ADF /NDF	0.450 ^b^ ± 0.0355	0.437 ^b^ ± 0.0143	0.694 ^a^ ± 0.0459	0.399 ^bc^ ± 0.0249	0.475 ^b^ ± 0.0153	0.656 ^a^ ± 0.0366	0.604 ^a^ ± 0.0214	0.466 ^b^ ± 0.0256	0.333 ^c^ ± 0.0085	0.402 ^bc^ ± 0.0418	0.001
CP ^7^	89.0 ^d^ ± 7.70	167 ^c^ ± 3.7	140 ^c^ ± 4.3	269 ^ab^ ± 28.1	176 ^c^ ± 5.0	129 ^cd^ ± 20.3	242 ^b^ ± 42.5	311 ^a^ ± 10.3	352 ^a^ ± 49.8	202 ^bc^ ± 31.3	0.001

^a–e^ Fisher means test (α = 0.05). ^1^ Species: corn silage (CS); mombasa grass (*Megathyrsus maximus*; MOM); Black Mulberry (*Morus nigra*; BMU); Aroeira (*Lithraea molleoides*; ARO); Candeia (*Eremanthus erythropappus*; CAN); Gliricídia (*Gliricidia sepium*; GLI), Santa Bábara tree (*Melia azedarach*; SBT), Tithonia (*Tithonia diversifolia*; TIT), and White Mulberry (*Morus alba*; WMU). ^2^ *p*: probability of specie effect. ^3^ DM: dry matter (g/kg as-fed). ^4^ OM: organic matter (g/kg DM). ^5^ NDF: neutral detergent fiber (g/kg DM). ^6^ ADF: acid detergent fiber (g/kg DM). ^7^ CP: crude protein (g/kg DM).

**Table 2:** In vitro degradation and gas production from edible trees and shrubs in Brazil.

Item	Species ^1^										*p* ^2^
	CS	MOM	ARO	BMU	CAN	JAT	GLI	SBT	TIT	WMU	
In vitro degradation, g/kg											
DM ^3^	578 ^d^ ± 42.7	557 ^d^ ± 21.8	598 ^d^ ± 15.9	777 ^ab^ ± 22.3	329 ^f^ ± 17.1	461 ^e^ ± 19.0	709 ^c^ ± 21.2	775 ^b^ ± 12.4	806 ^a^ ± 24.3	819 ^a^ ± 10.0	<0.001
NDF ^4^	334 ^ab^ ± 10.1	418 ^a^ ± 30.4	65 ^cd^ ± 43.4	392 ^ab^ ± 47.2	29 ^d^ ± 17.2	93 ^c^ ± 10.2	230 ^bc^ ± 58.8	180 ^c^ ± 39.8	495 ^a^ ± 75.5	447 ^a^ ± 46.4	<0.001
pH	6.43 ^bc^ ± 0.068	6.40 ^bc^ ± 0.076	6.33 ^c^ ± 0.088	6.68 ^ab^ ± 0.184	6.94 ^ab^ ± 0.021	6.93 ^a^ ± 0.019	6.27 ^c^ ± 0.314	6.54 ^b^ ± 0.089	6.81 ^a^ ± 0.087	6.27 ^c^ ± 0.059	0.002
In vitro gas production model											
A ^5^	113 ^bc^ ± 2.60	143 ^ab^ ± 17.4	87.8 ^cd^ ± 7.94	112 ^bc^ ± 27.2	72.8 ^d^ ± 5.37	79.8 ^d^ ± 6.48	114 ^b^ ± 5.0	158 ^a^ ± 8.3	117 ^bc^ ± 40.4	162 ^a^ ± 7.5	0.006
B ^6^	0.05 ± 0.008	0.05 ± 0.004	0.07 ± 0.006	0.06 ± 0.010	0.06 ± 0.005	0.05 ± 0.009	0.05 ± 0.010	0.05 ± 0.007	0.03 ± 0.012	0.07 ± 0.012	0.387
C ^6^	−0.19 ± 0.059	−0.15 ± 0.020	−0.30 ± 0.045	−0.18 ± 0.064	−0.20 ± 0.036	−0.11 ± 0.058	−0.12 ± 0.085	−0.09 ± 0.089	−0.11 ± 0.069	−0.21 ± 0.078	0.581
T ^7^	4.09 ± 1.184	1.93 ± 0.467	3.67 ± 1.15	2.82 ± 0.845	2.47 ± 0.100	1.03 ± 0.422	1.96 ± 0.967	4.21 ± 0.936	2.35 ± 1.536	2.53 ± 0.522	0.429
GP48 ^8^	83.3 ^cd^ ± 3.70	107 ^bc^ ± 14.9	72.0 ^d^ ± 7.60	94.3 ^cd^ ± 23.25	59.2 ^e^ ± 5.42	65.8 ^de^ ± 5.35	93.0 ^c^ ± 4.76	128 ^ab^ ± 12.2	72.9 ^d^ ± 7.75	143 ^a^ ± 7.2	0.008
24 h- methane	2.81 ^b^ ± 0.250	3.59 ^bc^ ± 0.34	1.56 ^b^ ± 0.099	3.57 ^b^ ± 1.127	1.84 ^b^ ± 0.423	2.65 ^b^ ± 0.475	3.88 ^bc^ ± 1.102	5.60 ^c^ ± 1.361	0.11 ^a^ ± 0.010	6.18 ^c^ ± 0.412	<0.001

^a–e^ Fisher means test (α = 0.05). ^1^ Species: corn silage (CS); mombasa grass (*Megathyrsus maximus*; MOM); Black Mulberry (*Morus nigra*; BMU); Aroeira (*Lithraea molleoides*; ARO); Candeia (*Eremanthus erythropappus*; CAN); Gliricídia (*Gliricidia sepium*; GLI), Santa Bábara tree (*Melia azedarach*; SBT), Tithonia (*Tithonia diversifolia*; TIT), and White Mulberry (*Morus alba*; WMU). ^2^ *p*: probability of specie effect. ^3^ DM: dry matter. ^4^ NDF: neutral detergent fiber. ^5^ A is potential gas production (mL/g dry matter). ^6^ b and c are parameters of fractional gas production. ^7^ T is the *lag time* (h). ^8^ GP48 is gas production at 48 h of incubation.

## Data Availability

The datasets are with authors and will be available in the MDPI section.
